# Development and validation of a method for the simultaneous analysis of fatty acid ethyl esters, ethyl sulfate and ethyl glucuronide in neonatal meconium: application in two cases of alcohol consumption during pregnancy

**DOI:** 10.1007/s00216-021-03248-0

**Published:** 2021-03-23

**Authors:** Mateusz Kacper Woźniak, Laura Banaszkiewicz, Justyna Aszyk, Marek Wiergowski, Iwona Jańczewska, Jolanta Wierzba, Agata Kot-Wasik, Marek Biziuk

**Affiliations:** 1 Department of Forensic Medicine, Faculty of Medicine, Medical University of Gdańsk, 3A Marii Skłodowskiej-Curie Str., 80-210 Gdańsk, Poland; 2 Department of Analytical Chemistry, Faculty of Chemistry, Gdańsk University of Technology, 11/12 Narutowicza Str., 80-233 Gdańsk, Poland; 3 Pharmaceutical Plant Polpharma SA, 19 Pelplińska Str., 83-200 Starogard Gdański, Poland; 4 Department of Neonatology, The University Clinical Centre, Faculty of Medicine, Medical University of Gdańsk, 3A Marii Skłodowskiej-Curie Str., 80-210 Gdańsk, Poland; 5 Department of Internal and Pediatric Nursing, Faculty of Health Sciences with Institute of Maritime and Tropical Medicine Institute of Nursing and Midwifery, Medical University of Gdańsk, 3A Marii Skłodowskiej-Curie Str., 80-210 Gdańsk, Poland

**Keywords:** Ethyl glucuronide, Ethyl sulfate, Fatty acid ethyl esters, GC-MS, Meconium, LC-MS/MS

## Abstract

Alcohol consumption during pregnancy constitutes one of the leading preventable causes of birth defects and neurodevelopmental disorders in the exposed children. Fatty acid ethyl esters (FAEEs), ethyl glucuronide (EtG) and ethyl sulfate (EtS) have been studied as potential biomarkers of alcohol consumption. However, most analytical approaches proposed for their analysis in meconium samples consist of separated extraction procedures requiring the use of two meconium aliquots, which is costly in terms of both time and materials. Therefore, the aim of this study was to develop and validate a method for the simultaneous extraction of 9 FAEEs, EtG and EtS from one meconium aliquot. The sample was homogenized using methanol, and then FAEEs were extracted with hexane while EtG and EtS were isolated using acetonitrile. Then, extracts were applied to solid-phase extraction columns and analysed by gas chromatography mass spectrometry (FAEEs) and liquid chromatography tandem mass spectrometry (EtG and EtS). Calibration curves were linear with *r* values greater than 0.99. The LODs ranged from 0.8 to 7.5 ng/g for FAEEs and were 0.2 ng/g and 0.8 ng/g for EtS and EtG, respectively. LOQs ranged from 5 to 25 ng/g for FAEEs and were 1 ng/g and 2.5 ng/g for EtS and EtG, respectively. Accuracies and precisions were between 93.8 and 107% and between 3.5 and 9.7%, respectively. The recovery values ranged from 89.1 to 109%. The method proved to be sensitive, specific, simple and fast and allowed for the reduction of the amount of organic solvent used for extraction compared to other published data while higher recoveries were obtained. The method was used for analysis of meconium samples in two cases of mothers who were consuming alcohol during pregnancy.

**Supplementary Information:**

The online version contains supplementary material available at 10.1007/s00216-021-03248-0.

## Introduction

Excessive use of ethyl alcohol constitutes a significant problem and causes many medical conditions. Instances of aggressive behaviour, sexual assaults, family problems and car accidents caused by individuals under the influence of alcohol are still commonplace. Prevention and removal of these effects is very costly for governments, as well as for the healthcare system [1, 2]. The consumption of alcohol during pregnancy is of great concern because it has a toxic impact not only on the mother but also on the foetus. Ethanol easily crosses the placental barrier, putting the foetus at risk of developmental problems, body defects, mental retardation and neurodevelopmental disorders. A wide range of foetal abnormalities were named foetal alcohol spectrum disorder (FASD). Importantly, the dose-effect correlation between maternal alcohol consumption and the development of FASD is not well understood and it is estimated that even low amounts of ethanol may adversely affect foetal and infant growth [3, 4]. Therefore, reliable recognition of alcohol consumption during pregnancy is crucial to protect the health of children but the currently used methods include mainly self-reported maternal questionnaires, which lacks in sensitivity and reliability.

Most ingested ethanol is metabolized in the liver via oxidation while only a small percentage undergoes non-oxidative processes, resulting in the formation of fatty acid ethyl esters (FAEEs; 0.1%), ethyl glucuronide (EtG; 0.6–1.5%) and ethyl sulfate (EtS; 0.1%). All of these listed non-oxidative metabolites are biomarkers of alcohol consumption. Determination of such compounds in biological materials can solve the problem of the poor sensitivity of methods utilizing questionnaires [2]. Many samples have been utilized for the detection of alcohol biomarkers, but in the last decade, neonatal meconium screening has increased in popularity for the recognition of prenatal exposure to drugs of abuse. This is due to the relatively simple and non-invasive collection of this material. Meconium is the first neonatal stool, and its formation starts between the 12th and 16th weeks of gestation. Therefore, meconium analysis extends the window of detection and provides more information than urine or blood for the detection of intrauterine drug exposure. Most newborns (approximately 70%) excrete meconium in the first 12 h of life, 93% within the first 24 h and 99.8% during the first 48 h [5, 6].

Due to the different physicochemical properties of FAEEs (non-polar compounds) and EtG and EtS (both polar compounds) and the complex matrix composition of meconium samples, most analytical approaches for their analysis consist of separate extraction procedures requiring the use of two meconium aliquots. Moreover, procedures presented in the literature consist of many steps and require the use of a large volume of solvents for both liquid extraction and solid-phase extraction (SPE) to purify the obtained extracts. Other extraction methods, such as ultrasound-assisted liquid extraction or microwave-assisted extraction, have also been used for determinations, but each additional step makes the procedure more complicated, expensive and time consuming. For chromatographic separation, gas chromatography mass spectrometry (GC-MS) and/or liquid chromatography tandem mass spectrometry (LC-MS/MS) were used [1, 7]. To date, only one paper describing the simultaneous extraction of FAEEs (9 compounds), EtG and EtS has been published [4]. However, the authors developed a procedure consisting of many steps. Purification of the extracts after liquid extraction was also required; this purification step was performed using two different SPE columns. This approach extended the process time and increased the use of organic solvents for conditioning of the SPE sorbents and elution of the analytes. Moreover, low extraction recoveries were obtained (as low as 51–62% for FAEEs and 66% for EtS), except for EtG (97%). Two injections into the LC-MS/MS system (separate analysis of FAEEs and EtS/EtG) were required for chromatographic separation of the analytes. Another method presented in the literature allows for simultaneous extraction of only 4 FAEEs and EtG with a long sample sonication time requiring acetonitrile (as long as 15 min) [3]. Therefore, to the best of our knowledge, there are no methods for the simultaneous extraction of all of the above-mentioned non-oxidative biomarkers from meconium samples and the methods presented in the literature can be improved, simplified and shortened.

Based on the above information, the aim of this study was to develop and validate a fast and simple method for the simultaneous extraction of 9 FAEEs, EtG and EtS in one meconium aliquot using a single SPE column. We focused on shortening the extraction time and reducing the amount of organic solvent required for liquid extraction and SPE, as well as for chromatographic separation. Therefore, for FAEE analysis, GC-MS was applied instead of LC-MS/MS. GC should be also the first choice because LC is a source of pollutants (organic solvents used as the mobile phase). For EtG and EtS determinations, the use of GC-MS was not possible without derivatization, which is not recommended in terms of green chemistry. We also focused on obtaining high extraction recoveries, which included lowering the detection and quantification limits.

## Materials and methods

### Chemicals

Individual certified standards of 9 FAEEs were obtained from Sigma-Aldrich (Merck, Warszawa, Poland). The standards were separately dissolved in hexane to obtain stock standard solutions at concentrations of 1 mg/mL. Individual methanolic–certified standard solutions of EtS (1 mg/mL), EtG (1 mg/mL), deuterated ethyl sulfate (EtS-D5; 1 mg/mL) and deuterated ethyl glucuronide (EtG-D5; 0.1 mg/mL) were purchased from Cerilliant Corporation (Round Rock, TX, USA). EE 17:0 was used as an internal standard (IS) for FAEE analysis. EtG-D5 and EtS-D5 were used as ISs for EtG and EtS analysis, respectively. Key information considered compounds included in the study with their abbreviations are listed in Table S1 in the Supplementary Information (ESM).

All solvents used were of HPLC grade. Methanol (MeOH) and acetonitrile (ACN) were supplied by Merck (Warszawa, Poland), and hexane, dichloromethane (DCM) and ethyl acetate were obtained from Sigma-Aldrich (Merck, Warszawa, Poland). Analytical-grade aqueous ammonium hydroxide (NH_3_) at a concentration of 25% and formic acid (FA) were acquired from POCH S. A. (Avantor Performance Materials Poland S.A., Gliwice, Poland) and Merck (Warszawa, Poland), respectively. Water was purified by a Millipore Milli-Q Gradient A10 water purification system (Merck, Warszawa, Poland). CHROMABOND^®^ NH_2_ aminopropyl–modified silica weak anion-exchange SPE columns (100 mg/1 mL, 200 mg/3 mL and 500 mg/3 mL; pore diameter: 55–75 Å; particle size: 20–50 μm; surface area: >360 m^2^/g) were purchased from MACHEREY-NAGEL (Avantor Performance Materials Poland S.A., Gdańsk, Poland).

Four solutions used for conditioning, washing and elution of analytes during SPE were prepared. Their compositions are presented in the section “SPE”. These solutions were prepared freshly prior to each extraction to maintain the integrity of their properties due to the high volatility of ammonium hydroxide and FA, which changes the composition of solutions during storage.

### Meconium samples

Meconium samples were obtained from babies born in Gdańsk University Clinical Centre, Clinic of Neonatology, Poland. Meconium was scraped a using plastic spatula from diapers as soon as possible after birth, placed in plastic vials (Falcons) and deep frozen at −20 °C (to avoid degradation of analytes) prior to delivery to the Gdańsk University of Technology to perform sample preparation. Instrumental analyses were performed at the Medical University of Gdańsk (GC-MS) and at the Gdańsk University of Technology (LC-MS/MS).

Several samples of neonatal meconium were collected in cases where mothers proved that they did not drink alcohol during pregnancy and were used as a blank sample throughout the validation process. Additional confirmation that this blank sample was free of analytes was obtained by chromatographic analysis (compounds of interest were below limits of detection (LODs)).

### Stock solutions, calibrators and quality control (QC) samples

Appropriate volumes of individual FAEE stock standard solutions were mixed together and diluted with hexane to obtain FAEE working solutions at concentrations of 100 μg/mL, 10 μg/mL and 1 μg/mL. A 10 μg/mL IS working solution for FAEE determination (IS-FAEE working solution) was prepared by dilution of the stock standard solution of EE 17:0 with hexane. Stock solutions of EtS/EtG (as a mixture) were obtained by dilution of certified standard solutions to concentrations of 10 μg/mL, 1 μg/mL and 0.1 μg/mL by MeOH. A 2 μg/mL IS mixture of EtS-D5 and EtG-D5 (IS-EtS-EtG stock solution) was also prepared by dilution with MeOH and was used for the determination of EtS and EtG. All solutions were stored in amber glass vials at −20 °C until use and were prepared freshly each week.

Calibrators (number of replicates, *n* = 3) were prepared by spiking 200 mg of meconium sample with appropriate amounts of corresponding FAEE working solutions and stock solutions of EtS/EtG to obtain concentrations of 2.5 ng/g, 5 ng/g, 25 ng/g, 50 ng/g, 100 ng/g, 250 ng/g, 500 ng/g, 1000 ng/g and 2500 ng/g and 1 ng/g, 2.5 ng/g, 5 ng/g, 10 ng/g, 25 ng/g, 50 ng/g, 100 ng/g, 250 ng/g, 500 ng/g and 1000 ng/g meconium for FAEE and EtS/EtG determinations, respectively. Subsequently, extraction was performed, followed by chromatographic analysis.

QC samples (*n* = 3) were prepared for each analyte at three concentration levels (listed in Table S3 in the ESM) across the linear dynamic range by spiking blank meconium samples with analytes and the ISs, similar to the calibrators. QC samples were used to assess accuracy and precision of the method.

### Sample preparation

**Fig. 1:**
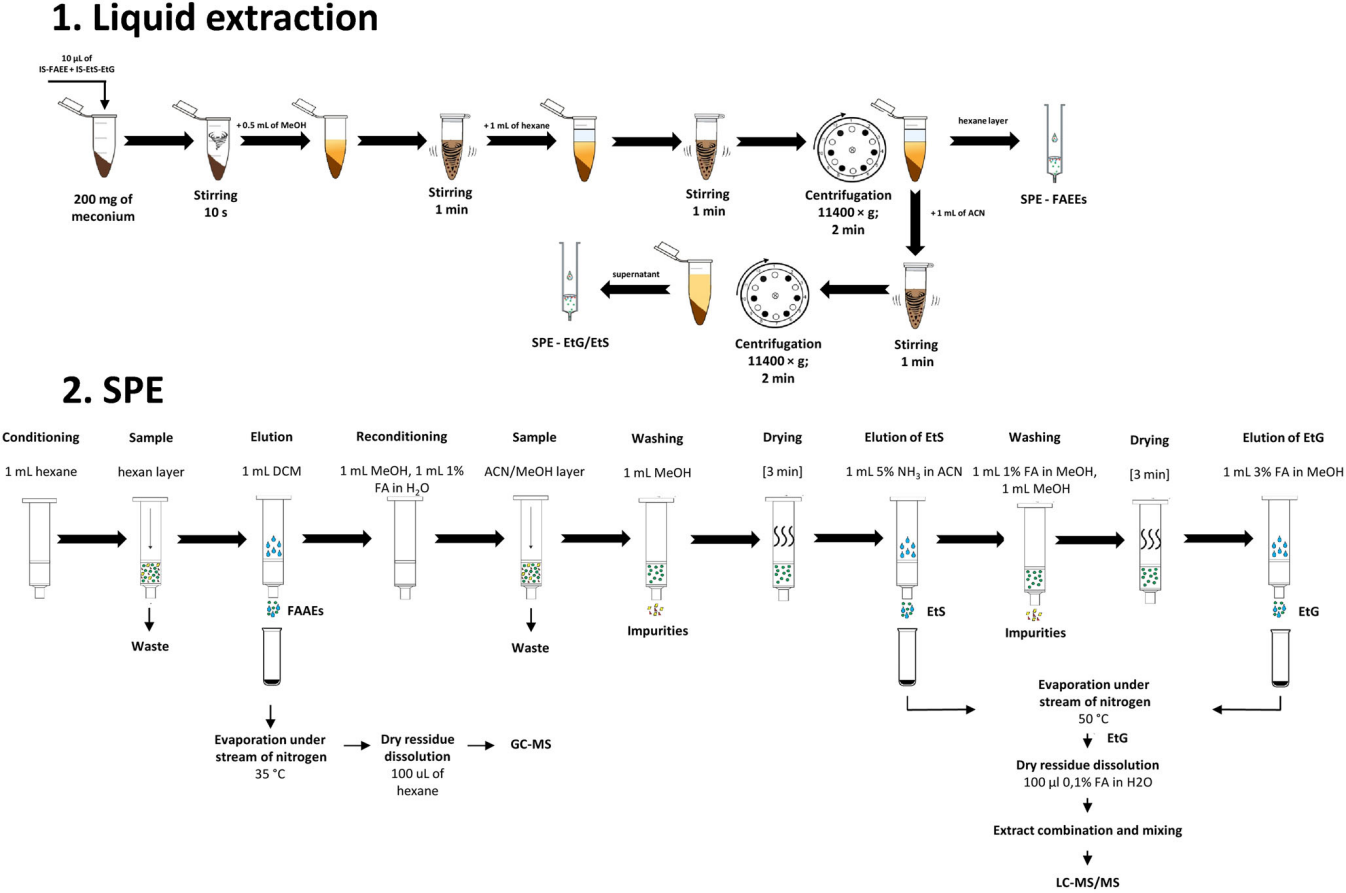
Workflow of the analytical procedure applied for the determination of FAEEs, EtG and EtS in meconium samples

Graphical workflow for analytical procedure developed in this study is presented in Fig. 1.

#### Liquid extraction

Meconium was allowed to thaw for approx. 1 h at room temperature and was homogenized by manual mixing using a spatula. Then, an aliquot of 200 ± 5 mg of meconium was weighed into a 2-mL Eppendorf tube followed by the addition of 10 μL of IS-FAEE working solution and IS-EtS-EtG stock solution, and the sample was vortexed for 10 s. Subsequently, 0.5 mL of MeOH was added, and the meconium was homogenized by vortexing for 1 min. After the addition of 1 mL of hexane, the sample was vortexed for 1 min to extract FAEEs and centrifuged at 11,400×*g* for 2 min. Then, the upper layer (hexane) was directly transferred to an SPE column after conditioning for FAEE determination. To the remaining residue of meconium sample containing MeOH, 1 mL of ACN was added, and the sample was vortexed for 1 min and centrifuged at 11,400×*g* for 3 min. Then, a supernatant was directly transferred to an SPE column after conditioning to isolate EtG and EtS.

#### SPE

Each SPE column was conditioned by 1 mL of hexane. Then, the supernatant (hexane) after liquid extraction was passed through the SPE cartridge. Elution of FAEEs was performed using 1 mL of DCM into a glass vial. Subsequently, the SPE column was reconditioned by 1 mL of MeOH followed by 1 mL of a solution of FA in water (1:99, *v*/*v*). Then, the second supernatant from liquid extraction (ACN/MeOH layer) was applied (drying of the sorbent was not required). The SPE column was washed with 1 mL of MeOH, and high vacuum was applied for 3 min to dry the sorbent. EtS was eluted using 1 mL of a solution of ammonium hydroxide in ACN (5:95, *v*/*v*) into a new glass vial. Then, impurities were removed from the sorbent using 1 mL of a solution of FA in MeOH (1:99, *v*/*v*) and 1 mL of MeOH followed by drying using a high vacuum for 3 min. EtG was eluted using 1 mL of a solution of FA in MeOH (3:97, *v*/*v*) into another glass vial. Subsequently, all extracts were evaporated under a stream of nitrogen (FAEEs: 35 °C, EtG and EtS: 50 °C). The dry residue was dissolved in 100 μL of appropriate solvent or solution, in hexane (FAEEs) and in 0.1% FA/water (*v*/*v*; EtG). Then, the solution containing EtG was transferred to a glass vial containing the dry EtS residue, and the sample was vortexed. All extracts were then transferred to inserts placed in autosampler vials and chromatographically analysed.

Importantly, a substantial loss of FAEEs was observed during the evaporation step after the solvent was removed. This loss was due to the high volatility of FAEEs. Therefore, to avoid losses of these compounds by evaporation, the nitrogen stream was stopped immediately after the solvent was removed.

### Instrumentation

#### GC-MS conditions

FAEE determinations were achieved using a 7890B GC System (gas chromatograph) coupled to a 5977B single quadrupole mass spectrometer with an electron impact (EI) ionization source (Agilent Technologies). The GC-MS instrument was equipped with a split/splitless CIS 4 (cooled injection system) injection system allowing for temperature programming of the injection port and a multi-purpose sampler (MPS) robotic autosampler (Gerstel GmbH & Co. KG). The temperature of the injector was initially set at 110 °C and after 5 s was increased to 260 °C at 10 °C/s, which was held until the end of the analysis. Pulsed splitless mode for 1 min with the initial injection pressure set at 40 psi for 0.5 min was used. Subsequently, split mode (20:1) was applied. The separation of analytes was carried out on a Phenomenex ZB-5 MS capillary column (30 m × 0.25 mm id and 0.25 μm film thickness; Shim-pol, Izabelin, Poland) with helium at a purity of 99.999% as the carrier gas in a constant flow of 1 mL/min. The GC oven temperature was programmed at 100 °C, increased to 200 °C at 25 °C/min, increased to 230 °C at 5 °C/min and finally increased to 300 °C at 25 °C/min. Post-run conditioning was carried out for 3 min at 300 °C. The temperatures of the MS transfer line, ion source and quadrupole were set at 285 °C, 230 °C and 150 °C, respectively. The MS was operated in positive mode (electron energy of 70 eV). For the identification and quantification of the analytes, selective ion monitoring (SIM) mode was applied with the ions listed in Table 1. The injection volume was 2 μL. GC data acquisition and quantification were accomplished using MassHunter GC/MS Acquisition software (version B.07.05.2479) and MassHunter Quantitative Analysis for GCMS software (version B.08.00) by Agilent Technologies and Maestro 1 software by Gerstel GmbH & Co. KG (version 1.5.3.2/3.5).

**Table 1:** Parameters of the GC-SIM-MS system used for quantification of FAEEs

Detection window		Analyte	Rt (min)	Ions (*m*/*z*)*			Relative ion area ratio
No.	Rt range (min)						
1	4–5.5	EE 12:0	5.01	**88**	101	157	100/50/15
2	5.5–7	EE 14:0	6.35	**88**	101	157	100/55/20
3	7–8.7	EE 16:0	8.17	**88**	101	157	100/59/18
4	8.7–9.6	IS	9.21	**88**	101	157	100/62/19
5	9.1–11	EE 18:2	10.11	**81**	95	109	100/75/40
		EE 18:1	10.17	**88**	101	264	100/90/60
		EE 18:3	10.20	**79**	108	306	100/40/4
		EE 18:0	10.42	**88**	101	157	100/60/20
6	11–11.7	EE 20:4	11.62	**91**	105	119	100/60/45
7	11.7–12.8	EE 20:0	11.98	**88**	101	157	100/65/25

*Quantifying ions are given in bold

#### LC-MS/MS conditions

Analysis of EtS and EtG was performed using the ultra-performance liquid chromatography (UPLC) Nexera X2 system (Shimadzu, Japan) comprising a DGU-20A5R degasser, a CBM-20A controller, an LC-30AD binary pump, an SIL-30AC autosampler and a CTO-20AC column oven. Separation was achieved on a Phenomenex Luna^®^ Omega Polar C18 column (100 mm × 2.1 mm, 3 μm pore size) equipped with a Polar C18 SecurityGuard™ Cartridge guard cartridge (4 mm × 2 mm, 3 μm pore size) (Shim-pol, Izabelin, Poland). The column temperature was maintained at 30 °C, the flow rate was kept at 0.4 mL/min and the injection volume was 10 μL. The mobile phase used for the separation consisted of water with 0.1% FA (*v*/*v*; component A) and ACN with 0.1% FA (*v*/*v*; component B). Chromatographic separation was performed in gradient elution mode as follows: 0 min (0% B), 1 min (0% B) and 5 min (90% B) kept for 3 min. Then, the initial column conditions were restored over 4 min.

The detection system consisted of an LCMS-8060 triple quadrupole mass spectrometer (Shimadzu, Japan) equipped with an electrospray ionization source operated in negative (ESI−) multiple reaction monitoring (MRM) mode. The parameters of the ion source were set as follows: nebulizing gas flow, 3 L/min; heating gas flow, 10 L/min; interface temperature, 300 °C; DL temperature, 250 °C; heat block temperature, 400 °C; and drying gas flow, 10 L/min. The capillary voltage was at −3 kV. Data acquisition and quantification were accomplished using LabSolutions v5.85 software. The optimum detection conditions are presented in Table 2.

**Table 2:** MS/MS parameters with retention times for compounds analysed by LC

Analyte	Rt (min)	Precursor ion (*m*/*z*)	Product ion (*m*/*z*)*	Collision energy (V)	Q1 prerod (V)	Q3 prerod (V)
EtS	1.15	125.00	**96.90**	20	14	23
			80.15	31	12	18
EtS-D5	1.13	130.00	**98.00**	19	19	18
			80.15	34	23	18
EtG	2.48	221.00	**75.10**	17	23	28
			85.15	16	10	12
EtG-D5	2.39	226.05	**75.05**	15	16	12
			84.95	18	11	28

*MRM values used for quantification are given in bold

### Method validation

Method validation experiments were conducted according to the procedure published by the Scientific Working Group for Forensic Toxicology (SWGTOX) [8] and other data in the field of our study [9]. Selectivity; linearity; carry-over effects; matrix effects (MEs); sensitivity, in terms of LODs and limits of quantification (LOQs); precision; accuracy (both intra- and inter-day assays); recovery; and batch stability were evaluated over validation. Criteria for the identification of analytes were retention time (Rt), the presence of three characteristic ions (or two MRM transitions) and their abundance ratios. Rt should be no more than ±0.1 min compared to standards while ion ratio difference maximum of 20% compared to standards was adopted.

#### Selectivity

Ten meconium samples (considered blank samples) obtained from mothers who proved that they did not drink alcohol during pregnancy were analysed. The presence of peaks of endogenous interferences at the retention times of analytes and ISs was evaluated (intensities of such peaks should be below the LODs). Exogenous interferences as common pharmaceuticals and illicit drugs were also evaluated by fortifying these compounds into blank meconium samples at a concentration of 1000 ng/g (*n* = 6). These interferences consisted compounds from the various groups, i.e. analgesic and inflammatory drugs, amphetamine-type stimulants, benzodiazepines, cannabinoids, opioids and their metabolites. List of these compounds is given in Table S2 in the ESM.

#### Linearity

The linearity was verified as the correlation coefficient (*r*) of the calibration curves constructed after analysis of the calibrators.

#### Carry-over effects

To evaluate the carry-over caused by sample processing, extracts of blank matrix samples were processed immediately after the injection of samples containing analytes at a concentration equal to the highest calibrator and to five times the highest calibrator. This test was performed 6-fold.

#### MEs

Due to the potential variability in matrix composition obtained from different sources, MEs were investigated using 6 samples screened first to be negative for the analytes. ME studies were performed using the post-extraction addition technique. In this experiment, two sets of solutions of analytes and ISs (*n* = 3) were prepared at three concentration levels (as shown in Table S3 in the ESM): in hexane (GC-MS analysis) or in the mobile phase (LC-MS/MS analysis) (set A) and in matrix extracts obtained from meconium samples (set B). The MEs were calculated using the following formula: MEs = ((*A*_set B_/*A*_set A_) − 1) × 100%, where *A*_set B_ and *A*_set A_ are the ratio of peak areas for analytes and IS in set B and set A, respectively.

#### Sensitivity

The LODs and LOQs were evaluated. The LODs were calculated as the concentration giving a signal-to-noise ratio (*S*/*N*) equal to 3 (peak-to-peak noise definition) for the lowest ions or MRM transitions of each compound. LOQs generally correspond to the lowest point on the calibration curves due to linearity, but the requirement of an *S*/*N* ratio equal to a minimum 10 was also verified.

#### Accuracy and precision

Intra- and inter-day assay accuracy and precision were evaluated by analysing QC samples. The accuracy was calculated as the mean ratio of the measured concentrations and the nominal concentration. Then, the analyses were repeated over 3 consecutive days to evaluate the inter-day assay accuracy and precision as between-day averages (fresh QC samples were prepared each day). The precision was calculated as the coefficient of variation (CV) of these measurements.

#### Recovery

The analyte-to-IS peak area ratios of the spiked and extracted blank meconium samples were compared with the corresponding analyte-to-IS peak area ratios of the appropriate matrix extracts fortified with standards (*n* = 3). The recoveries were investigated at 3 concentration levels, similar to the accuracy and precision experiment. In these experiments, IS solutions were added post-extraction to avoid loss during the extraction step.

#### Batch stability

Batch stability was measured by injecting QC samples maintained in the autosampler at 4 °C (LC-MS/MS) and at room temperature (RT; GC-MS) at the beginning of the run sequence and after 24 h.

## Results and discussion

### Method development

#### GC-MS and LC-MS/MS analyses

Due to the various physicochemical properties of analytes, two instrumental techniques were incorporated in the study: GC-MS and LC-MS/MS for FAEE and EtS/EtG quantification, respectively.

**Fig. 2:**
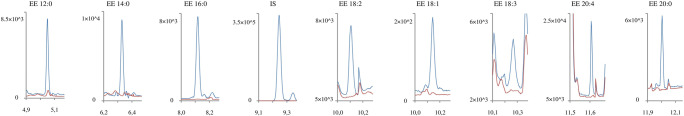
GC-SIM-MS chromatograms for the FAEE blank meconium sample (red line) and blank meconium sample spiked with analytes at concentrations corresponding to the lowest calibrator level (blue line). Only quantifier ions are shown

All required GC-MS parameters were optimized. Initially, determination of retention times for analytes, optimization of the oven temperature gradient and gas flow rate to obtain chromatographic separation in a short analysis time and selection of characteristic ions for each compound were performed. These experiments were performed in full-scan acquisition (SCAN) mode (scan range 40–450 *m*/*z*). For most FAEEs, chromatographic separation was achieved (peak resolution Rs > 1.5), except for 3 compounds: EE 18:1, 18:2 and 18:3. However, it was possible to select specific ions for these unresolved compounds; thus, chromatographic separation was not required. Problems with chromatographic separation of these compounds can be explained by their similar structure, as they are isomers; hence, achieving separation of these compounds is difficult. Three ions for each analyte were selected, and SIM mode was implemented. Subsequently, optimization of other chromatographic conditions, such as the injector temperature rate, as well as the injection mode was performed. The total method run time was 15.8 min (including post-run conditioning) with a data acquisition time of 8.8 min. Chromatograms for FAEEs are presented in Fig. 2 (blue line).

MRM optimization was performed before LC analyses. In this experiment, flow injection analysis (FIA) mode was used. The most intensive ion was produced by losing a proton [M − H]^−^ in negative mode for all analytes. Each precursor ion was then fragmented in the collision cell, and two specific and the most intense product ions were selected to create the MRM transitions for each analyte. Subsequently, all voltages and *m*/*z* values were optimized automatically by LabSolutions software. All further analyses were performed in MRM mode. To increase the sensitivity, optimization of MS source parameters, such as nebulizing gas flow, heating gas flow, interface temperature, DL temperature, heat block temperature and drying gas flow, was performed.

**Fig. 3:**
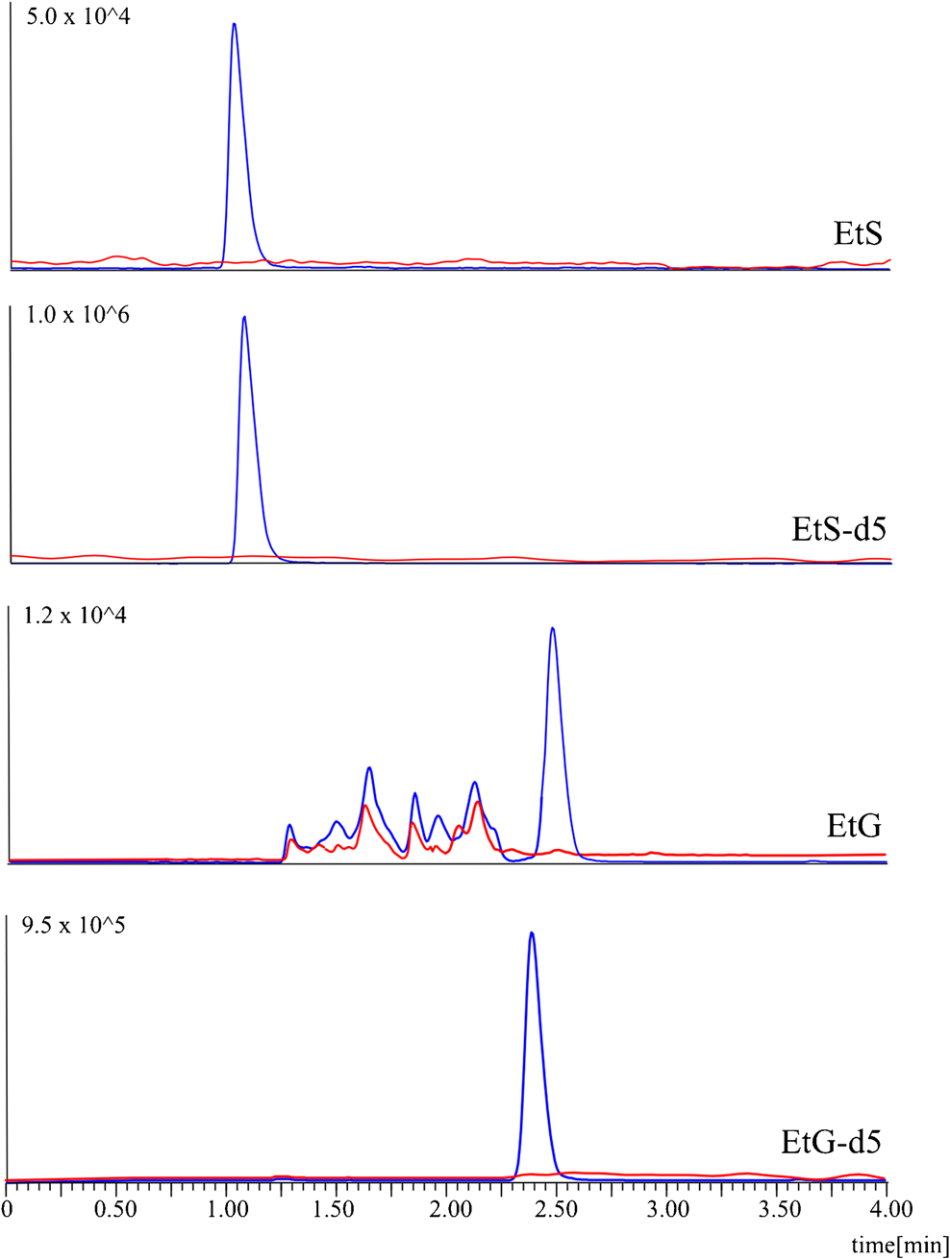
LC-MRM-MS/MS chromatograms of the blank meconium sample (red line) and blank meconium sample spiked with analytes at concentrations corresponding to the lowest calibrator level (blue line). Only quantifier MRMs are shown

During LC-MS/MS method development, a series of experiments were performed to obtain narrow peaks, separation of analytes from the co-extracted matrix components, a short analysis time and good sensitivity. However, achieving an optimal retention factor (*k*) or at least *Rt* longer than the dead time of the chromatographic column is problematic in reversed-phase liquid chromatography (RPLC) due to the high polarity of EtG and EtS. Thus, in the first step, it was decided to use a Thermo Hypercarb™ column (50 mm × 2.1 mm; 3 μm) which allows to obtain good retention even for very polar analytes [10]. Although good peak shapes and satisfactory *k* factors were obtained for the analytes (Tables 3 and 4), after approximately 50 injections, we observed a loss in retention (drifting retention times), which was also stated in the literature [11]. To solve this problem, various washing procedures or backflushing should be performed. We tried such procedures, but drifting still occurred after the subsequent few injections. Therefore, we decided to use another chromatographic column (the final column) that allows for the application of water (100% component A as the mobile phase) for the initial gradient elution conditions to obtain retention of polar compounds. Good peak shapes and satisfactory *k* factors were obtained, so this column was selected for further analysis. The use of various buffers did not significantly influence the separation and peak shape or even decrease the MS signal, and wider peaks were obtained at high buffer concentrations. Finally, a mixture of water and ACN (both with the addition of 0.1% FA (*v*/*v*)) in gradient mode was selected as the mobile phase. Chromatograms for EtS and EtG with corresponding ISs are presented in Fig. 3 (blue line).

**Table 3:** Chosen separation conditions of the LC-MS/MS system for the two tested columns

Column	Thermo Hypercarb™ column (50 mm × 2.1 mm; 3 μm)	Phenomenex Luna^®^ Omega Polar C18 column (100 × 2.1 mm; 3 μm)
Flow rate (mL/min)	0.6	0.4
Column temperature (°C)	40	30
Injection volume (μL)	5	10
Analysis time (min)	11	13
Mobile phase components	A: H_2_O + 0.1% FA (*v*/*v*) B: ACN + 0.1% FA (*v*/*v*)	
Gradient elution	0–1 min, 10% B 1–5 min, 90% B 5–7 min, 90% B	0–1 min, 0% B 1–5 min, 90% B 5–9 min, 90% B

**Table 4:** Comparison of the selected chromatographic parameters

Chromatographic parameters	EtS/EtS-D5	EtG/EtG-D5	EtS/EtS-D5	EtG/EtG-D5
Rt (min)	1.23/1.21	0.60/0.59	1.15/1.13	2.48/2.39
*k*	5.15/5.05	2.00/1.95	1.13/1.09	3.59/3.43
TF (10%)	1.01/1.05	1.21/1.17	1.15/1.21	1.01/1.05
*w*_50%_ (min)	0.075/0.074	0.047/0.047	0.074/0.074	0.081/0.079

*k* retention factor, *Rt* retention time, *TF (10%)* tailing factor at 10% of height, *w*_*50%*_ width at 50% of peak height

#### Optimization of the extraction procedure

Our preliminary study showed that extracts from meconium samples obtained by liquid extraction contain many impurities that co-elute with the analytes; thus, proper quantification of target compounds is impossible. Therefore, a clean-up step must be performed before chromatographic analysis. Many authors have suggested using an SPE with aminopropyl-modified silica-based sorbent for effective cleaning extracts after liquid extraction from meconium samples for FAEE analysis. However, in the present literature, various sorbent masses and types and volumes of solvents are used [3, 4, 7, 12]. Therefore, in the first step, optimization of the parameters of the SPE procedure was performed. Aminopropyl-modified silica weak anion-exchange SPE columns (CHROMABOND^®^ NH_2_) containing sorbent masses equal to 100 mg, 200 mg and 500 mg were tested in model studies. The SPE columns were conditioned using 1 mL (100 mg and 200 mg sorbent mass) and 2 mL (500 mg sorbent mass) of hexane, and then model solutions were applied (1 mL of solution of all FAEEs in hexane). Subsequently, FAEEs were eluted five times using 1 mL of hexane, and eluates were collected into separate glass vials (each consisting of separate fractions). Model solutions passed through the SPE sorbent were also collected as first fractions. IS-FAEE working solution was added to each vial, the solutions were evaporated under a stream of nitrogen at 35 °C and the dry residues were dissolved in hexane and analysed. Recoveries in % (*R*) were calculated to assess extraction efficiency (peak areas for analytes vs. peak areas for the IS after and before SPE). Elution profiles are presented in Fig. S1 in the ESM. In all cases, recoveries were higher than 94%, but the collection of the following number of fractions was required:

- For 100 mg of sorbent: the second and third fractions;
- For 200 mg of sorbent: the second, third and fourth fractions; and
- For 500 mg of sorbent: the second, third, fourth and fifth fractions.

In the next step, all required fractions for each SPE column were collected into one vial to assess significant differences between tested sorbents (*n* = 3). The *F* test and Student’s *t* test (*α* = 0.05) were used to compare the results in terms of precision (standard deviation (SD)) and mean values, respectively. All investigated SPE methods did not differ statistically in terms of precision. Subsequently, there were also no statistically significant differences observed for the obtained mean values for recoveries between SPE columns containing 100 mg and 200 mg of sorbent. However, there were statistically significant differences in the mean recoveries for a few compounds for SPE columns containing 500 mg of sorbent (100 mg vs. 500 mg and 200 mg vs. 500 mg). The lowest recoveries were obtained for the 500 mg SPE columns. Taking into account also volume of solvent required to elute analytes, the first column was chosen for further study. The results are presented in Fig. S2 in the ESM. Subsequently, various solvents (DCM, MeOH and ethyl acetate) were tested for elution of FAEEs from the SPE column selected as previously described. Each sorbent was conditioned using 1 mL of hexane, and a model solution of FAEE was applied. Further steps were performed as described for the previous experiment. Elution profiles are presented in Fig. S3 in the ESM. Furthermore, all required fractions for each SPE column were collected into one vial to assess significant differences between tested solvents (*n* = 3). The highest recoveries were obtained for hexane and DCM (above 90%), with no statistically significant differences. However, using DCM, only 1 mL of solvent was sufficient to elute FAEEs in comparison to hexane in which case 2 mL of solvent was required (Fig. S4 in the ESM).

For the clean-up of meconium extracts after liquid extraction before chromatographic separation of EtS and EtG, various types of sorbents are suggested [1, 3, 4, 13, 14]. The polar properties of EtG and EtS as well as the presence of isoelectric points in the structure of EtG cause difficulties with selective trapping of these compounds on the SPE sorbent and their subsequent elution. An additional problem consists of the removal of impurities co-extracted from meconium samples from the SPE sorbent with a simultaneous lack of analyte loss (as described below). Therefore, several different SPE sorbents were evaluated during the model study, including CHROMABOND^®^ NH_2_ (the same as in the case of FAEEs), Strata™-X-AW polymeric weak anion exchanger, Strata^®^ NH_2_ polymeric weak anion exchanger and Strata-X-A polymeric strong anion exchanger sorbents. Various solvents, mixtures of solvents, solvents at different pH values (acidic and alkaline) and buffers for conditioning and elution of analytes were tested (data not shown). In these experiments, methanolic solutions of EtG and EtS (1 mL) at a concentration of 20 ng/mL (similar to during optimization of SPE for FAEE determination) were used as model solutions. The highest recoveries (96% for EtS and 98% for EtG) were obtained for the CHROMABOND^®^ NH_2_ column (100 mg sorbent mass) when elution was carried out using 1 mL of a mixture of ammonium hydroxide with MeOH (5:95, *v*/*v*).

Based on the physicochemical properties of the tested compounds and paper published by Himes et al. [4], we decided to extract FAEEs using non-polar solvents and EtS and EtG using polar solvents from meconium samples. However, meconium samples are in a solid form, and it is recommended to homogenize them before liquid extraction (MeOH or ACN is suggested for this purpose). Our preliminary study showed that by using MeOH, better homogenization of meconium can be achieved versus using ACN. Therefore, initially, we decided to perform liquid extraction as follows: a 200-mg aliquot of meconium was homogenized with 0.5 mL of MeOH followed by extraction of FAEE using 1 mL of hexane. After removing the hexane layer, the residue was mixed with 0.5 mL of ACN to isolate EtS and EtG. Both extracts were applied to the SPE column because matrix composition can influence SPE efficiency compared to model studies, and some washing steps were added to the procedure in this case. Although the SPE procedure for FAEE determination optimized during the model study was sufficient to clean up the supernatants, MeOH/ACN extracts still contained many impurities, and the procedure did not sufficiently remove large matrix interferences affecting EtG and EtS. Therefore, many other experiments were performed to optimize the SPE procedure; many new solutions and combinations of published methods were evaluated, including different loading conditions, further sorbent washing with various solvents and different elution parameters, to test whether a single SPE approach could be achieved. The most problematic process was to wash the sorbent without elution of analytes because only strong basic or acidic solutions of organic solvents were able to remove impurities, but the analytes were also eluted. A mixture of ammonium hydroxide with ACN (5:95, *v*/*v*) allowed for selective elution of EtS, while impurities and EtG were retained on the sorbent. We also found that a mixture of FA in MeOH (5:95, *v*/*v*) was able to elute impurities, but EtG was also eluted. Therefore, we tested solutions of MeOH containing various amounts of FA to determine whether it is possible to selectively remove impurities without EtG and further elute EtG. Finally, a solution of FA in MeOH (1:99, *v*/*v*) allowed the removal of impurities, but only a solution of FA in MeOH (3:97, *v*/*v*) eluted EtG. Importantly, eluates of EtS and EtG should be collected in separate vials due to the formation of salts (ammonium formate), which lead to a decrease in the MS signal. Both eluates were combined after the evaporation step. In these experiments, recoveries were between 92 and 102% for FAEEs and between 45 and 60% for EtG and EtS. Therefore, various polar solvents (including MeOH, ACN, isopropanol and water) were added to the remaining meconium aliquot after extraction of FAEEs to improve the extraction efficiency of EtG and EtS. In all experiments, various volumes of solvents were tested to obtain high recoveries and low MEs. The use of 1 mL of ACN for extraction showed the highest recoveries and effectively removed solid particles suspended in MeOH after homogenization.

In summary, a CHROMABOND^®^ NH_2_ SPE column with a sorbent mass equal to 100 mg using a combination of solvents and solution at alkaline and acidic pH values (for conditioning, washing and elution) showed the best extraction yield of analytes, the lowest chromatographic interference and the lowest MEs. Importantly, only one SPE column was used.

### Method validation

The validation data are summarized in Table 5 and Table S3 in the ESM. There was no evidence of carry-over. Four out of 10 potential blank samples analysed during the selectivity test showed few compounds at a level above the LODs. Such findings were obvious due to the formation of alcohol biomarkers from endogenous alcohol. However, samples used for the development and validation of the method did not contain compounds of interest (chromatograms of blank sample are presented in Fig. 2 and Fig. 3; red line). No peaks of potential exogenous interferences were present in the retention times of analytes. Additionally, none of these interferences caused failure in ion/transition ratio or quantification criteria. QC samples maintained in the LC and GC autosamplers during the batch stability test were stable for 24 h (8–10% loss of analytes). Both enhancement and suppression of the MS signal were observed. MEs varied from slight signal suppression to high signal enhancement, and significant values were obtained for most compounds (only MEs between −20 and 20% are considered negligible); thus, matrix-matched calibration was performed instead of external calibration. The CVs for MEs between different sources of blank matrix did not exceed 15%. Due to the different properties of analytes and the use of two analytical techniques, various MS responses were obtained for the studied compounds. Therefore, different ranges of calibration curves were used. Calibration curves were constructed using the peak area ratio of analytes and appropriate IS vs. analyte concentrations. Weighted least square regression was applied to the calibration curves to improve the accuracy. Various weighting factors were verified, but the factor with the lowest sum of relative errors and the highest accuracy was selected for the compounds and was used for evaluation of the linearity, accuracy and precision. The method was shown to be linear within the tested ranges; the *r* values were all above 0.99. FAEE LODs ranged from 0.8 to 7.5 ng/g, while those for EtS and EtG were 0.2 ng/g and 0.8 ng/g, respectively. The LOQs were assumed to be the lowest points of the calibration curves and were between 5 and 25 ng/g for FAEEs, 1 ng/g for EtS and 2.5 ng/g for EtG. The accuracy and precision of the developed method were between 93.8 and 107% and between 3.5 and 9.7%, respectively. The recoveries ranged from 89.1 to 109% for all compounds. The CVs fulfilled the acceptance criteria (≤15%). All accuracy data were also within an acceptable range (between 85 and 115%); therefore, the international criteria for method validation in biomedical analysis/toxicology were fulfilled. The validation of the procedure demonstrated that the developed method is characterized by selectivity, high sensitivity and repeatability and can be used in the analysis of meconium samples in real cases.

**Table 5:** Calibration parameters

Analyte	Calibration range (ng/g)	Calibration curve equation	Weighting factor	LOD (ng/g)	*r*
EE 12:0	5–1000	*y* = 0.00212*x* + 0.0083	1/*x*	1.0	0.9993
EE 14:0	5–1000	*y* = 0.00231*x* + 0.0081	1/*x*^2^	0.8	0.9987
EE 16:0	5–1000	*y* = 0.00203*x* + 0.0075	1/*x*	1.2	0.9997
EE 18:2	25–2500	*y* = 0.00044*x* + 0.0068	1/*x*	7.5	0.9962
EE 18:1	10–1000	*y* = 0.00042*x* − 0.0002	1/*x*	2.5	0.9993
EE 18:3	10–2500	*y* = 0.00078*x* + 0.0003	1/*x*^2^	2.1	0.9960
EE 18:0	5–1000	*y* = 0.00182*x* + 0.0004	1/*x*	1.3	0.9994
EE 20:4	25–2500	*y* = 0.00021*x* + 0.0089	1/*x*^2^	7.4	0.9948
EE 20:0	10–1000	*y* = 0.00135*x* + 0.0004	1/*x*^2^	2.0	0.9976
EtS	1–1000	*y* = 0.02721*x* + 0.0227	1/*x*	0.2	0.9987
EtG	2.5–1000	*y* = 0.01140*x* + 0.0644	1/*x*	0.8	0.9981

### Analysis of real samples

The applicability of the developed method was proven by analysis of meconium samples obtained from two newborns in cases where mothers proved that they have consumed alcohol during pregnancy. Samples were prepared by the procedure and chromatographically analysed (*n* = 3). Results of the analysis are presented in Table 6.

**Table 6:** Determined concentrations of alcohol biomarkers (μg/g ± SD) in two positive cases with alcohol abuse

Case no.	EE 12:0	EE 14:0	EE 16:0	EE 18:2	EE 18:1	EE 18:3	EE 18:0	EE 20:0	Total FAEE	EtS	EtG
1	0.256 ± 0.018	<LOQ	0.355 ± 0.029	0.986 ± 0.077	2.36 ± 0.20	0.866 ± 0.092	0.756 ± 0.061	<LOQ	5.58	0.0329 ± 0.0031	1.77 ± 0.19
2	0.542 ± 0.045	1.12 ± 0.10	0.851 ± 0.042	2.41 ± 0.15	5.87 ± 0.41	0.241 ± 0.015	0.489 ± 0.075	0.0691 ± 0.0059	11.6	14.49 ± 0.95	22.9 ± 2.1

Both neonates had low birth weights and were small for gestational age (SGA). One of the neonates (from case no. 1) was a full-term neonate; for the 2nd pregnancy, which was of a female, the birth weight was below the 3rd percentile, while the body length was in the 90th percentile. During the first examination, the following facial features were visible: hypertelorism, smooth philtrum and a relatively short-upturned nose, prompting the physician to diagnose foetal alcohol syndrome (FAS). The mother had a history of one spontaneous abortion. She was 34 years old and unmarried and had a low educational level. She confessed alcohol consumption until the 20th week of pregnancy. The pattern of drinking was unknown. According to her self-report, she has since stopped drinking alcohol and started drug addiction treatment. The mother also smoked 20 cigarettes a day until the 20th week of pregnancy; later, she reduced the daily number of cigarettes she smoked to 6.

The 2nd neonate (case no. 2), a female, was born prematurely (36 weeks of gestation) at home and was the 5th pregnancy and 5th delivery of the mother, with birth weight in the 3rd percentile. Her mother was 36 years old, and she was also a tobacco smoker. The mother was admitted to the hospital and appeared to be drunk, with a high level of alcohol observed in the blood sample. The neonate, as the first neonate, demonstrated the typical FAS dysmorphic features.

High concentrations of alcohol biomarkers were determined in both cases in comparison to the literature (Table S4 in the ESM). However, there are no established and fully reliable cut-off values of alcohol biomarker (FAEEs, EtGs, EtSs) concentrations in meconium to differentiate heavy ethanol consumption from occasional maternal ethanol consumption during pregnancy or no use. However, based on the literature data and on ESM Table S4, it seems reasonable to employ cut-off limits of 0.6 μg/g FAEEs, 0.25 μg/g EtG and 0.0027 μg/g EtS for the assumption of binge drinking during pregnancy. Based on this assumption in case nos. 1 and 2 (Table 6), it was determined much higher concentrations of FAEEs, EtG and EtS than the cut-off values were determined.

### Comparison with other procedures

Initially, since 1994, only FAEEs were tested as potential biomarkers of alcohol consumption during pregnancy, and gas chromatography coupled to flame ionization detection (FID) or MS was used. The first developed procedures required the use of large amounts of solvents for extraction and a large meconium aliquot (1 g), and poor sensitivity was obtained. However, with the rapid development of more sensitive and selective instrumental techniques in the last decade (especially LC-MS/MS), the determination of EtG and EtS has gained importance because these biomarkers are present in meconium samples at low concentrations. Although the non-polar properties of FAEEs make them suitable for analysis by GC-MS, LC-MS/MS has recently been preferred for FAEE determination mainly due to the selectivity and sensitivity of this technique. Current studies are focused mainly on obtaining better sensitivity (lower LODs and LOQs), simplification, shortening of sample preparation step and reducing the use of solvents in these analytical methods [7, 15]. Due to many papers published on this topic, in Table S5 in the ESM, we present only the newest papers to compare them with the developed method. In brief, extraction of alcohol biomarkers is typically performed using two meconium aliquots and two separate SPE procedures or a combination of SPE with SPME. The sample mass used for analysis typically varies between 0.1 and 1 g [1, 3, 4, 7], except in procedures developed by Bakdash et al. [16] (10–20 mg for EtG quantification) and by Hutson et al. [17] (50 mg for FAEE quantification). Only one paper describing the simultaneous quantification of FAEEs, EtG and EtS has been published [4], but two SPE columns were used, while Vaiano et al. [3] developed a procedure with a single SPE column, but only 4 FAEEs and EtG were quantified. The presented method definitely stands out because we used only one meconium aliquot for extraction. Although a higher sample quantity (200 mg) was required than that in a previous study (100 mg), higher recoveries and lower LODs and LOQs were obtained with the use of a single SPE column, which definitely reduced the time and costs of analysis. The requirement of such a relatively larger meconium sample size was due to the use of GC-MS instead of LC-MS/MS, but GC is preferred whenever possible due to lower costs of purchase and maintenance and according to principles of green chemistry. Moreover, during liquid extraction, we either reduced the solvent volume or shortened the time (shorter mixing time). In Vaiano et al. [3], elution of EtG from an SPE column was performed using water, which, in next step, was evaporated over a long time. A similar approach was used by Bakdash et al. [16] and Tarcomnicu et al. [18]. In the presented method, solvent evaporation was performed within approximately 20–25 min. The method developed by Bakdash et al. [16] does not require a clean-up step of the extracts obtained by liquid extraction. However, as the authors stated, the main disadvantage of such an approach is obstruction of the precolumn by residues from the matrix, which had to be changed after approximately 200 injections. Separation at the chromatographic column also slowly worsened. Moreover, the recoveries, LODs and LOQs obtained in our study are also better than those in all previously published methods, while wider calibration ranges were achieved.

## Conclusion

Meconium analysis has gained popularity in recent years and provides an expanded time window for the detection of prenatal exposure to various substances, including alcohol. Through the simultaneous measurement of FAEE, EtG and EtS concentrations, it is possible to more accurately assess alcohol consumption during pregnancy compared to methods that allow for quantification of only one of these biomarkers or those based on maternal self-reported questionnaires. Methods used for meconium analysis are still being optimized in terms of sensitivity, extraction recoveries, shortened analysis times and operations according to the principles of green chemistry. The developed method in this study offers a significant reduction in both analytical costs and time and is characterized by good sensitivity and reproducibility for all biomarkers. This method is an environmentally friendly alternative method to others presented in the literature.

Although some information on cut-off values for alcohol biomarkers in meconium samples is available in the literature, further extended studies are needed to better understand the effect of biological variables (e.g. gender, age, metabolic diseases) on analysis, to establish correlations between daily alcohol consumption and the biomarker concentrations and to avoid false-positive and false-negative results. Therefore, this novel validated method for simultaneous extraction of FAEEs, EtG and EtS from one aliquot of meconium can be helpful for clinical and forensic applications for investigation of the best markers to identify in utero alcohol exposure.

## Supplementary information

ESM 1 (DOCX 105 kb)
