# Supplementary material for: Development and validation of a method for the simultaneous analysis of fatty acid ethyl esters, ethyl sulfate and ethyl glucuronide in neonatal meconium: application in two cases of alcohol consumption during pregnancy
Source: Anal Bioanal Chem. 2021 Mar 23;413(11):3093–105. doi: 10.1007/s00216-021-03248-0 (PMC8043868; doi:10.1007/s00216-021-03248-0)
Supplement: Supplementary file 1 — (DOCX 105 kb) [file 216_2021_3248_MOESM1_ESM.docx]

**Analytical and Bioanalytical Chemistry**

**Electronic Supplementary Material**

**Development and validation of a method for the simultaneous analysis of fatty acid ethyl esters, ethyl sulfate and ethyl glucuronide in neonatal meconium: Application in two cases of alcohol consumption during pregnancy**

Mateusz Kacper Woźniak^1,2,*^, Laura Banaszkiewicz^2^, Justyna Aszyk^2,3^, Marek Wiergowski^1^, Iwona Jańczewska^4^, Jolanta Wierzba^5^, Agata Kot-Wasik^2^, Marek Biziuk^2^

*^1^ Department of Forensic Medicine, Faculty of Medicine, Medical University of Gdańsk, 3A Marii Skłodowskiej-Curie Str., Gdańsk 80-210, Poland*

*^2^ Department of Analytical Chemistry, Faculty of Chemistry, Gdańsk University of Technology, 11/12 Narutowicza Str., Gdańsk 80-233, Poland*

*^3^ Pharmaceutical Plant Polpharma SA, 19 Pelplińska Str., Starogard Gdański 83-200, Poland*

*^4^ Department of Neonatology, The University Clinical Centre, Faculty of Medicine, Medical University of Gdańsk, 3A Marii Skłodowskiej-Curie Str., Gdańsk 80-210, Poland*

*^5^* *Department of Internal and Pediatric Nursing, Faculty of Health Sciences with Institute of Maritime and Tropical Medicine Institute of Nursing and Midwifery, Medical University of Gdańsk, 3A Marii Skłodowskiej-Curie Str., Gdańsk 80-210, Poland*

*Corresponding author*.E-mail address:* [*womat90@gmail.com*](mailto:womat90@gmail.com), mateusz.wozniak@gumed.edu.pl

Table S1 Key information considered compounds included in the study [1]

| **Compound name** | **Acronym** | **Structure** | **CAS No.** | **Molar mass** | **Log P** |
| --- | --- | --- | --- | --- | --- |
| Ethyl laurate;  Lauric acid ethyl ester | EE 12:0 |  | 106-33-2 | 228.20891 | 4.98 |
| Ethyl myristate; Myristic acid ethyl ester | EE 14:0 |  | 124-06-1 | 256.24021 | 5.87 |
| Ethyl palmitate;  Palmitic acid ethyl ester | EE 16:0 |  | 628-97-7 | 284.27151 | 6.76 |
| Ethyl heptadecanoate; Heptadecanoic acid etyl ester | EE 17:0 (IS) |  | 14010-23-2 | 298.28716 | 7.20 |
| Ethyl stearate; Stearic acid ethyl ester | EE 18:0 |  | 111-61-5 | 312.30281 | 7.65 |
| Ethyl oleate; Oleic acid ethyl ester | EE 18:1 |  | 111-62-6 | 310.28716 | 7.29 |
| Ethyl linoleate; Linoleic acid ethyl ester | EE 18:2 |  | [544-35-4](https://www.sigmaaldrich.com/catalog/search?term=544-35-4&interface=CAS%20No.&lang=en&region=US&focus=product) | 308.27151 | 6.93 |
| Ethyl linolenate; Linolenic acid ethyl ester | EE 18:3 |  | [1191-41-9](https://www.sigmaaldrich.com/catalog/search?term=1191-41-9&interface=CAS%20No.&lang=en&region=US&focus=product) | 306.25586 | 6.56 |
| Ethyl arachidate; Arachidic acid ethyl ester | EE 20:0 |  | 18281-05-5 | 340.33411 | 8.54 |
| Ethyl arachidonate; Arachidonic acid ethyl ester | EE 20:4 |  | 1808-26-0 | 332.27151 | 7.09 |
| Ethyl sulfate | EtS |  | 200-659-6 | 125.99867 | -0.11 |
| Ethyl glucuronide; Ethyl-β-D-glucuronide | EtG |  | 17685-04-4 | 222.07394 | -1.61 |

Table S2 List of exogenous interferences investigated in the study

| **Amphetamine-type stimulants** | **Sedative drugs** | **Cocaine and metabolites** | **Opioids** | **Others** |
| --- | --- | --- | --- | --- |
| methamphetamine, amphetamine, 3,4-metylenodioksyamfetamine, 3,4-metylenodioksymetamfetamine, 3,4-methylenedioxy-n-ethylamphetamine, phentermine, ephedrine, pseudoephedrine | zopiclone, 7-aminoclonazepam, 7-aminoflunitrazepam, zolpidem, chlordiazepoxide, midazolam, medazepam, flunitrazolam, zaleplon, estazolam, nitrazepam, oxazepam, alprazolam, lorazepam, clonazepam, triazolam, nordazepam, etizolam, flunitrazepam, flubromazepam, temazepam, tetrazepam, lormetazepam, phenazepam, diazepam, prazepam | cocaine, benzoylecgonine, cocaethylene, ecgonine methyl ester | buprenorphine, morphine, codeine, tramadol, 6-monoacetylmorphine, oxymorphone, oxycodone, dihydrocodeine, 2-ethylidene-1,5-dimethyl-3,3-diphenylpyrrolidine, hydromorphone, hydrocodone, buprenorphine, fentanyl, methadone, dextromethorphan | nicotine, cotinine, trans-3′-hydroxycotinine, Δ^9^-tetrahydrocannabinol (THC), 11-hydroxy-thc, 11-nor-9-carboxy-THC, caffeine, ketamine, norketamine, furosemid, acetaminophen, ibuprofen, 1-hydroxyibuprofen, 2-hydroxyibuprofen, 3-hydroxyibuprofen, carboxyibuproden, ketoprofen, acetylsalicylic acid, fluoxetine, gamma-hydroxybutyric acid, haloperidol, olanzapine, quetiapine, metoprolol, propofol |

| **a)** |  |
| --- | --- |
| **b)** |  |
| **c)** |  |

Fig. S1 Elution profiles of FAEEs during SPE optimization - comparison of the required fraction (1 mL each) to elute FAEEs from the SPE column: a) 100 mg, b) 200 mg, and c) 500 mg of sorbent. Hexane was used as the eluent

a

b

c

a

b

a

c

a

b

c

a

b

a

c

a

b

c

a

b

c

a

b

c

a

b

a

b

c

a

b

a

b

c

a

b

a

b

c

a

b

c

a

b

a

c

a

b

c

a

b

c

a

b

c

a

b

a

b

c

b

c

Fig. S2 Comparison of total recoveries of FAEEs (*n* = 3) for various masses of sorbent with the results of Student’s t-test. Data are expressed as the mean values ± SD. Bars with the same superscript (a, b, c) above refer to recoveries with no significant differences

| **a)** |  |
| --- | --- |
| **b)** |  |
| **c)** |  |

Fig. S3 Elution profiles of FAEEs during SPE optimization – comparison of the required fraction (1 mL each) to elute FAEEs from the SPE column (100 mg of sorbent) using various solvents: a) DCM, b) ethyl acetate, and c) MeOH. The elution profile for hexane is presented in Fig. S1 a

a

b

c

a

b

a

b

c

a

b

a

b

c

a

b

c

a

b

c

a

b

c

a

b

c

a

b

c

a

b

c

a

b

c

a

b

c

a

b

c

a

b

c

a

b

c

a

b

c

a

b

c

a

b

c

a

b

c

a

b

c

a

b

c

a

b

c

a

b

c

a

b

c

Fig. S4 Comparison of total recoveries of FAEEs (*n* = 3) using various elution solvents (100 mg of sorbent) with the results of Student’s t-test. Data are expressed as the mean values ± SD. Bars with the same superscript (a, b, c) above refer to recoveries with no significant differences. Data for MeOH were excluded because recoveries below 50% were obtained

**Table S3** Validation parameters of the developed method: accuracy (precision), matrix effects (CV) and recoveries

| Analytes | *C*  [ng/g] | Intra-dayassay [%] | | | Inter-dayassay [%] | ME  [%] | Recovery [%]  (mean ± SD) |
| --- | --- | --- | --- | --- | --- | --- | --- |
|  |  | Day 1 | Day 2 | Day 3 |  |  |  |
| EE 12:0 | 10 | 100 (2.9) | 100 (4.4) | 105 (5.9) | 102 (4.6) | –16 (4.4) | 102 ± 7.6 |
|  | 250 | 103 (6.6) | 102 (2.8) | 102 (6.6) | 102 (4.8) | –14 (5.9) | 96.8 ± 7.8 |
|  | 800 | 95.7 (0.9) | 99.1 (2.0) | 98.0 (6.1) | 97.6 (3.7) | –17 (3.1) | 103 ± 4.0 |
| EE 14:0 | 10 | 104 (2.6) | 101 (4.4) | 99.7 (4.5) | 102 (3.9) | –2.4 (2.1) | 109 ± 6.9 |
|  | 250 | 99.8 (5.3) | 98.4 (1.2) | 102 (7.2) | 99.9 (4.7) | –0.2 (6.4) | 96.3 ± 10.6 |
|  | 800 | 94.2 (4.5) | 97.0 (7.2) | 96.7 (6.9) | 96.0 (5.7) | –8.4 (10.1) | 103 ± 4.0 |
| EE 16:0 | 10 | 97.3 (3.3) | 100 (4.6) | 101 (2.9) | 99.5 (3.6) | 13 (2.1) | 100 ± 7.2 |
|  | 250 | 102 (3.7) | 100 (0.5) | 101 (8.4) | 101 (4.6) | 9 (5.6) | 94.2 ± 8.8 |
|  | 800 | 96.9 (2.6) | 99.3 (1.4) | 97.7 (8.3) | 98.0 (4.5) | –3.6 (8.2) | 103 ± 4.0 |
| EE 18:2 | 50 | 103 (8.1) | 109 (7.4) | 109 (4.7) | 107 (7.0) | 25 (7.1) | 100 ± 12 |
|  | 500 | 97.4 (2.3) | 98.6 (7.4) | 94.3 (5.2) | 96.8 (4.9) | 28 (6.9) | 105 ± 2.5 |
|  | 2000 | 99.8 (9.8) | 102 (2.9) | 96.0 (6.3) | 99.3 (6.5) | –2 (8.5) | 103 ± 4.0 |
| EE 18:1 | 25 | 98.2 (3.2) | 90.9 (7.6) | 95.7 (7.0) | 94.9 (6.6) | 34 (10.3) | 101 ± 5.1 |
|  | 250 | 98.0 (0.7) | 96.9 (5.8) | 95.7 (8.3) | 96.9 (5.2) | 11 (4.6) | 100 ± 9.6 |
|  | 800 | 99.2 (1.0) | 101 (5.0) | 105 (6.1) | 102 (9.7) | –5 (6.1) | 103 ± 4.0 |
| EE 18:3 | 25 | 98.9 (8.4) | 97.0 (2.2) | 95.7 (7.3) | 97.2 (5.9) | 43 (2.5) | 102 ± 9.6 |
|  | 500 | 106 (4.3) | 101 (7.9) | 95.7 (8.7) | 101 (7.5) | 5 (1.1) | 107 ± 1.8 |
|  | 2000 | 105 (3.8) | 98.2 (2.8) | 105(5.8) | 104 (4.2) | 33 (8.6) | 103 ± 4.1 |
| EE 18:0 | 10 | 100 (3.0) | 97.8 (5.7) | 96.6 (5.7) | 98.1 (4.7) | 26 (3.4) | 90.8 ± 3.8 |
|  | 250 | 102 (1.7) | 99.8 (2.5) | 96.6 (5.1) | 99.5 (3.8) | 13 (9.8) | 89.1 ± 5.8 |
|  | 800 | 94.7 (6.5) | 96.7 (2.7) | 105 (6.1) | 98.8 (6.6) | –2.6 (5.2) | 103 ± 4.0 |
| EE 20:4 | 50 | 98.3 (8.5) | 102 (11.3) | 99.9 (3.5) | 100 (7.5) | 66 (4.9) | 97.1 ± 8.5 |
|  | 500 | 94.2 (3.8) | 98.7 (3.8) | 99.4 (2.4) | 97.5 (3.9) | 22 (7.9) | 104 ± 8.7 |
|  | 2000 | 97.8 (6.3) | 98.8 (4.0) | 105 (6.1) | 101 (5.8) | 6 (4.6) | 103 ± 4.0 |
| EE 20:0 | 25 | 102 (7.8) | 98.5 (5.6) | 92.7 (2.2) | 97.8 (6.6) | 40 (5.1) | 90.1 ± 6.6 |
|  | 250 | 103 (0.4) | 97.4 (4.5) | 94.4 (2.0) | 98.1 (4.4) | 25 (6.3) | 90.7 ± 4.8 |
|  | 800 | 93.2 (6.3) | 98.1 (2.4) | 105 (6.1) | 98.8 (6.8) | 6 (8.5) | 103 ± 4.0 |
| EtS | 5 | 101 (6.7) | 114 (8.0) | 105 (9.3) | 104 (7.5) | –15 (1.1) | 90.2 ± 5.3 |
|  | 250 | 94.4 (3.3) | 98.9 (6.6) | 97.0 (4.0) | 97.9 (5.4) | 6 (2.6) | 92.5 ± 3.3 |
|  | 800 | 103 (0.9) | 103 (1.4) | 102 (6.7) | 102 (3.5) | –11 (3.4) | 95.6 ± 9.0 |
| EtG | 5 | 91.8 (6.6) | 91.2 (6.9) | 98.4 (11.7) | 93.8 (8.6) | 31(2.5) | 90.0 ± 8.5 |
|  | 250 | 95.8 (4.7) | 98.4 (6.9) | 105 (3.9) | 99.8 (6.2) | 9.3 (4.8) | 90.5 ± 8.9 |
|  | 800 | 101 (2.6) | 104 (5.3) | 107 (3.2) | 104 (4.1) | 3.1 (5.1) | 92.9 ± 8.9 |

**Table S4** Suggested cut-off values, range and median of concentrations of ethanol biomarkers (EtG, EtS, FAEE) in meconiumsamples

| **Biomarker** | **Mothers**  **(number of samples)** | **Cut-off** [nmol/g]** | **Range of concentrations [nmol/g]** | **Median of concentrations [nmol/g]** | **Ref.** |
| --- | --- | --- | --- | --- | --- |
| **EtG** | nondrinking (*n*=42)* | < 2  (< 0.44 µg/g) | 0.022-1.870 | 0.100 | [2] |
|  | nondrinking (*n*=52)* |  | 0.022-1.570 | 0.140 |  |
|  | uncertain exposure (*n*=23)* |  | 0.022-0.650 | 0.160 |  |
|  | uncertain exposure (*n*=36)* |  | 0.040-10.500 | 0.250 |  |
|  | certain exposure (*n*=5) |  | 2.510-95.720 | 7.240 |  |
|  | drinking (*n*=49) | ≥ 1.5  (≥ 0.33 µg/g) | 4.1-49.0 | n/a | [3] |
|  | drinking (*n*=49) |  | 4.1-55.1 | n/a |  |
|  | drinking (*n*=96) | not established | 5.0-796.2 [ng/g] | 15.6 [ng/g] | [3] |
|  | drinking (*n*=81) |  | 6.8-6309.3 [ng/g] | 101.5 [ng/g] |  |
| **EtS** | nondrinking (*n*=8)* | not established | 0.008-0.020 | 0.010 | [2] |
|  | nondrinking (*n*=15)* |  | 0.010-0.030 | 0.020 |  |
|  | uncertain exposure (*n*=12)* |  | 0.010-0.520 | 0.020 |  |
|  | uncertain exposure (*n*=7)* |  | 0.010-0.110 | 0.020 |  |
|  | certain exposure (*n*=5) |  | 0.014–0.235 | 0.330 |  |
|  | drinking (*n*=49) | ≥ 0.012  (≥ 0.0016 µg/g) | 0.0-26.5 | n/a | [3] |
|  | drinking (*n*=49) |  | 6.1-14.3 | n/a |  |
|  | drinking (*n*=96) | not established | 1-65.2 [ng/g] | 15.6 [ng/g] | [4] |
|  | drinking (*n*=81) |  | 1.1-437.5 [ng/g] | 101.5 [ng/g] |  |
| **FAEE ***** | drinking (*n*=4)* | ≥ 2  (≥ 0.6 µg/g) | 0.200-0.370 | 0.290 | [2] |
|  | drinking (*n*=18)* | ≥ 2  (≥ 0.6 µg/g) | 0.240-66.020 | 2.460 |  |
|  | drinking (*n*=96) | ≥ 2  (≥ 0.6 µg/g) | 2.8-3.5 | n/a | [4] |
|  | drinking (*n*=81) | ≥ 2  (≥ 0.6 µg/g) | 2.2-324.7 | n/a |  |
|  | binge drinking (review article) | ≥ 0.6 µg/g | n/a | n/a | [5] |

* Only samples with measurable concentration (> LLOQ) of EtG and EtS were considered for this calculation.

** To compare values with the results obtained in this study, units in nmol were recalculated for µg; 1 nmol of EtG and EtS correspond to 0.22 and 0.13 µg, respectively. Due to various mass of individual FAEE, it is assumed that 1 nmol of FAEE is calculated as 0.3 µg.

***Different FAEE compounds were determined by different authors as total FAEE.

**Table S5** Comparison of published methods for the determination of FAEEs, EtG and EtS in meconium samples

| Analytes | Sample mass [g] | Extraction | Clean-up step | Chromatography | Recoveries [%] | LOD/LOQ [ng/g] | Ref |
| --- | --- | --- | --- | --- | --- | --- | --- |
| FAEEs (9), EtG, EtS* | 0.2 | Liquid  (2.5 mL) | SPE (8 mL) | GC-MS and LC-MS/MS | 89.1-109 | FAEE: 0.8-7.5/5-25  EtS: 0.2/2.5  EtG: 0.8/2.5 | Thisstudy |
| FAEEs (9), EtG, EtS* | 0.1 | Liquid  (3.8 mL) | double SPE (15 mL) | LC-MS/MS | 51.2-96.5 | FAEE: 15-50/25-50  EtG/EtS: 2.5-5/2.5-5 | [6] |
| FAEEs (4), EtG | 0.2 | Liquid (2 mL) + sonication 15min | SPE (6 mL) | LC-MS/MS | 75-93 | FAEE: 15-15/15-15  EtG: 7/10 | [7] |
| FAEEs (4) | 0.05 | Homogenization with buffer | SPME | GC-MS | 2.9-11.7 | 15-48/39-96** | [8] |
| EtG | 0.01-0.02 | Liquid (0.5 mL) + shaking 20 min | filtration over regenerated cellulose | LC-MS/MS | 86.6-106 | 10-30 | [9] |
| EtG | 0.2 | Liquid (1 mL) + sonication 10 min | SPE (2 mL+unknown volume of hexane) | LC-MS/MS | 38-40 | -/50 | [10] |
| EtG, EtS | 0.2 | Liquid (1 mL) + sonication 10 min | SPE (3 mL) | LC-MS/MS | 78.7-96.8 | EtG: 1.5/5  EtS: 0.3/1 | [11] |
| FAEEs (9) | 1 | Liquid (6 mL) + mixing 10 min | SPE (3 mL) | LC-MS/MS | 53.6-98.3 | 11.3-15.9/29.3-51.2 | [12] |
| FAEEs (4) | 0.5 | Liquid (5 mL) | SPME | GC-MS | 3.2-19.5 | 6.3-11.9/18.8-35.8 | [13] |
| FAEEs (9) | 0.5 | Liquid (6 mL) + mixing 10 min | SPE (7.5 mL) | LC-MS/MS | 40-86 | 3-24/6-81** | [14] |

* simultaneous extraction of analytes; in other presented studies FAEEs were determined separately then EtG or EtS

** in the paper values were given in nmol/g, but to clarify data, values were converted using mean mass of FAEE 300 ng/g [5]

In column named “Extraction” and “Clean-up step” in brackets were listed amounts of organic solvents used; water was excluded as relatively low cost of its use and that it is considered as environmentally friendly solvent.

**References**

1. https://www.chemicalize.com/welcome. Accessed 30 Nov 2020.

2. Morini L, Groppi A, Marchei E, Vagnarelli F, Algar OG, Zuccari P, et al. Population baseline of meconium ethyl glucuronide and ethyl sulfate concentrations in newborns of nondrinking women in 2 mediterranean cohorts. Ther Drug Minit. 2010;32:359–63. https://doi.org/10.1097/FTD.0b013e3181d5f14a.

3. Morini L, Marchei E, Vagnarelli F, Garcia Algar O, Groppi A, Mastrobattista L, et al. Ethyl glucuronide and ethyl sulfate in meconium and hair-potential biomarkers of intrauterine exposure to ethanol. Forensic Sci Int. 2010;196:74–7. https://doi.org/10.1016/j.forsciint.2009.12.035.

4. Pichini S, Morini L, Marchei E, Palmi I, Rotolo MC, Vagnarelli F, et al. Ethylglucuronide and ethylsulfate in meconium to assess gestational ethanol exposure: preliminary results in two Mediterranean cohorts. Can J Clin Pharmacol. 2009;16(2):e370-5.

5. dos Santos FS, de Martinis BS, Furtado EF. The detection of fetal alcohol exposure by FAEEs meconium analysis. Curr Dev Disord Reports. 2016;3:325–241. https://doi.org/10.1007/s40474-016-0102-x.

6. Himes SK, Concheiro M, Scheidweiler KB, Huestis MA. Validation of a novel method to identify in utero ethanol exposure: Simultaneous meconium extraction of fatty acid ethyl esters, ethyl glucuronide, and ethyl sulfate followed by LC-MS/MS quantification. Anal Bioanal Chem. 2014;406:1945–1955. https://doi.org/10.1007/s00216-013-7600-z.

7. Vaiano F, Favretto D, Palumbo D, Cooper G, Mactier H, Busardò FP, Mari F, Bertol E (2016) A novel, simultaneous extraction of FAEE and EtG from meconium and analysis by LC-MS/MS. Anal Bioanal Chem 408:2587–2594 . https://doi.org/10.1007/s00216-016-9364-8

8. Hutson JR, Aleksa K, Pragst F, Koren G. Detection and quantification of fatty acid ethyl esters in meconium by headspace-solid-phase microextraction and gas chromatography-mass spectrometry. J Chromatogr B. 2009;877:8–12. https://doi.org/10.1016/j.jchromb.2008.10.056.

9. Bakdash A, Burger P, Goecke TW, Fasching PA, Reulbach U, Bleich S, et al. Quantification of fatty acid ethyl esters (FAEE) and ethyl glucuronide (EtG) in meconium from newborns for detection of alcohol abuse in a maternal health evaluation study. Anal Bioanal Chem. 2010;396:2469–77. https://doi.org/10.1007/s00216-010-3474-5.

10. Tarcomnicu I, van Nuijs ALN, Aerts K, De Doncker M, Covaci A, Neels H. Ethyl glucuronide determination in meconium and hair by hydrophilic interaction liquid chromatography-tandem mass spectrometry. Forensic Sci Int. 2010;196:121–7. https://doi.org/10.1016/j.forsciint.2009.12.043.

11. Morini L, Marchei E, Pellegrini M, Groppi A, Stramesi C, Vagnarelli F, et al. Liquid chromatography with tandem mass spectrometric detection for the measurement of ethyl glucuronide and ethyl sulfate in meconium: new biomarkers of gestational ethanol exposure? Ther Drug Monit. 2008;30:725–32. https://doi.org/10.1097/FTD.0b013e31818b2fd9.

12. Pichini S, Pellegrini M, Gareri J, Koren G, Garcia-Algar O, Vall O, et al. Liquid chromatography-tandem mass spectrometry for fatty acid ethyl esters in meconium: Assessment of prenatal exposure to alcohol in two European cohorts. J Pharm Biomed Anal. 2008;48:927–33. https://doi.org/10.1016/j.jpba.2008.07.026.

13. Hutson JR, Rao C, Fulga N, Aleksa K, Koren G. An improved method for rapidly quantifying fatty acid ethyl esters in meconium suitable for prenatal alcohol screening. Alcohol 2011;45:193–9. https://doi.org/10.1016/j.alcohol.2010.07.005.

14. Kwak HS, Kang YS, Han KO, Moon JT, Chung YC, Choi JS, et al. Quantitation of fatty acid ethyl esters in human meconium by an improved liquid chromatography/tandem mass spectrometry. J Chromatogr B. 2010;878:1871–4. https://doi.org/10.1016/j.jchromb.2010.05.001.
